# Sodium-Glucose Cotransporter-2 Inhibitor in Diabetic and Nondiabetic Renal Transplant Recipients

**DOI:** 10.1016/j.ekir.2024.11.033

**Published:** 2024-11-28

**Authors:** Lucie Maigret, Lucile Basle, Valérie Chatelet, Laure Ecotiere, Peggy Perrin, Léonard Golbin, Dominique Bertrand, Dany Anglicheau, Coralie Poulain, Cyril Garrouste, Clément Danthu, Charlotte Boud'hors, Yannick Le Meur, Manon Dekeyser, Fabien Duthe, Bénédicte Sautenet, Pierre-Guillaume Deliège, Philippe Gatault

**Affiliations:** 1 Service de Néphrologie-Hypertension artérielle, Dialyses, Transplantation rénale, CHRU de Tours, Tours, France; 2 Service de Néphrologie, Dialyse et Transplantation, CHU de Reims, Reims, France; 3 Service de Néphrologie et Transplantation Rénale, CHU de Caen, Caen, France; 4 Service de Néphrologie et Transplantation Rénale, CHU de Poitiers, Poitiers, France; 5 Service de Néphrologie, Dialyse et Transplantation, CHU de Strasbourg, Strasbourg, France; 6 Service de Néphrologie et Transplantation Rénale, CHU de Rennes, Rennes, France; 7 Service de Néphrologie et Transplantation Rénale, CHU de Rouen, Rouen, France; 8 Département de Néphrologie et transplantation rénale, Hôpital Necker Enfants Malades, Assistance Publique-Hôpitaux de Paris, Paris, France; 9 Service de Néphrologie et Transplantation Rénale, CHU d’Amiens, Amiens, France; 10 Service de Néphrologie et Transplantation Rénale, CHU de Clermont-Ferrand, Clermont-Ferrand, France; 11 Service de Néphrologie et Transplantation Rénale, CHU de Limoges, Limoges, France; 12 Service de Néphrologie et Transplantation Rénale, CHU d’Angers, Angers, France; 13 Service de Néphrologie et Transplantation Rénale, CHU de Brest, Brest, France; 14 Service de Néphrologie, CHU d’Orléans, Orléans, France; 15 Unité INSERM UMR 1327 ISCHEMIA, Tours, France

**Keywords:** proteinuria, renal transplantation, sodium-glucose cotransporters-2 inhibitors

## Abstract

**Introduction:**

Sodium-glucose cotransporter-2 inhibitors (SGLT2i) improve cardiovascular prognosis in patients with chronic kidney disease (CKD), diabetes, and heart failure; and slow the decline of kidney dysfunction in patients with albuminuria. Although safety and efficacy of SGLT2i have not been investigated in kidney transplant recipients (KTRs), their marketing authorization leaves the possibility of their use in these patients in France.

**Methods:**

This was a prospective multicenter real-life study including all consecutive KTRs treated with SGLT2i.

**Results:**

We identified 347 KTRs treated with SGLT2i (97% with dapagliflozin), with an initiation of treatment most often beyond the first year after transplantation (87%). Importantly, 226 (65.1%) were diabetic and 245 (70.6%) were treated with angiotensin-converting enzyme (ACE) inhibitors or angiotensin-receptor blockers (ARBs). We found a low incidence of urinary tract infections (UTIs) (6.6%) and genital mycosis (0.6%), without any serious adverse event. Overall, SGLT2i were stopped in 54 patients (15.6%). The causes of SGLT2i discontinuations were very diverse. The main causes were graft dysfunction (32%), intercurrent infections (17%), urinary infections (11%), and digestive symptoms (9%). KTRs with a low estimated glomerular filtration rate (eGFR), especially those with eGFR < 30 ml/min per 1.73 m^2^, presented with the highest incidence of SGLT2i discontinuation (*P* = 0.003). SGLT2i were associated with a reduction in proteinuria, found in both diabetic and nondiabetic KTRs. In addition, they had an antihypertensive effect restricted to uncontrolled-hypertensive patients.

**Conclusion:**

SGLT2i have been used in KTRs since their authorization in France. They were discontinued more frequently in patients with impaired graft function; however, the expected side effects were infrequent and not life-threatening. The short-term antiproteinuric and antihypertensive effects are promising.

## Graphical abstract

**Figure:**
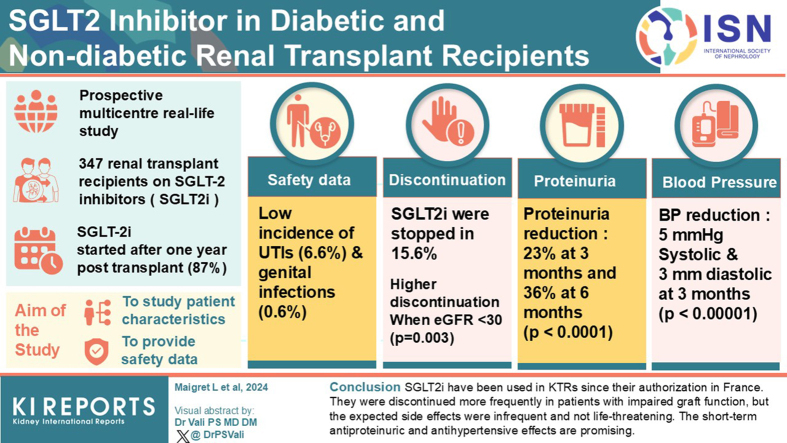

See Commentary on Page 660

SGLT2i are essential nephroprotective agents in patients with CKD. Indeed, dapagliflozin and empagliflozin slow the progression of CKD in patients with albuminuria when used as an adjunctive therapy in combination with ACE inhibitors or ARBs, regardless of diabetic status.^1^^,^^2^ Moreover, cardiovascular mortality is decreased in patients with albuminuria having CKD, treated with dapagliflozin.^1^^,^^3^ Therefore, SGLT2i are recommended by Kidney Disease Improving Global Outcomes, American Diabetes Association, and the European Association for the Study of Diabetes as first-line therapy in patients with type 2 diabetes and coexisting or high-risk conditions for heart failure, CKD, or atherosclerotic cardiovascular disease.^4^, ^5^, ^6^ They are also recommended for patients with heart failure, regardless of ejection fraction and diabetes status, as well as for patients with diabetes.^7^^,^^8^ As a result, SGLT2i are widely prescribed by nephrologists, diabetologists, cardiologists, and general practitioners.

Although KTRs have been excluded from randomized clinical trials, some of their characteristics would legitimize the use of SGLT2i. First, proteinuria is frequent in KTRs and is strongly associated with an increased risk of death and graft loss.^9^^,^^10^ Second, the increasing prevalence of pretransplant diabetes, driven by global epidemiology and the high prevalence of posttransplant diabetes mellitus are associated with poor patient and graft outcomes.^11^, ^12^, ^13^ Third, chronic cardiac failure is frequently observed in KTRs because of the high prevalence of coronary artery disease and hypertension. It can also be underscored that elderly recipients with numerous comorbidities, often transplanted with extended criteria donor, are particularly susceptible to developing heart failure with preserved ejection fraction.^14^

Since 2019, a few case series and small comparative studies released have investigated the use of SGLT2i in diabetic KTRs with stable graft function. These studies primarily examined the use of empagliflozin that seemed not to be associated with serious adverse events. A large retrospective case-controlled cohort study from South Korea investigated the safety and efficacy of SGLT2i in diabetic KTRs, suggesting that cardiovascular and nephroprotective effects may be similar to those observed in patients with CKD, with a good safety profile.^15^ These findings were corroborated by a Spanish multicenter observational study, which found reductions in blood pressure and proteinuria, and noted a slightly increased risk of UTIs, especially in female recipients and patients with a history of UTI.^16^

In France, dapagliflozin was approved for use in adults with CKD and the inclusion criteria of the DAPA-CKD trial not excluding of KTRs opened the way to its use in this population. We therefore designed a prospective multicenter observational study to monitor the prescription of SGLT2i in this population, with the aim of reporting early safety and efficacy data.

## Methods

### Study Design

GREAT-ASTRE (Gliflozin in Renal Transplantation) is an observational study that targeted to enroll all patients treated with SGLT2i in all 13 centers of the Spiesser group. Patients consented to participate in the ASTRE database that prospectively collects clinical, biological, and histological data in more than 10,000 KTRs (DR-2012-518). Visits to ASTRE were scheduled on the day of transplantation, at 3 months, 1 year, then annually. We added 3 database visits: initiation of SGLT2i, then at 3 and 6 months. We collected the following data: diabetes, weight, systolic and diastolic blood pressure, creatinine, proteinuria (g/d or protein-to-creatinine ratio), hemoglobin A1C in diabetic patients; as well as treatments related to hypertension, dyslipidemia, and diabetes.

### Selection of Patients

We aimed to include all KTRs that started SGLT2i between June 2021 and March 2023, without any exclusion criteria except those with an unknown date of initiation of treatment. This was a real-life observational study, and each center was free to decide its own clinical practice. We did nevertheless collectively agree in a scientific meeting to avoid initiation of SGLT2i in the first 3 months following the transplantation, in patients with a history of numerous UTIs and with ureteral or bladder catheter. Data on the few patients treated with SGLT2i before June 2021 were retrospectively collected.

### Objectives

Our main objective was to describe the characteristics of patients treated with SGLT2i in real-life and provide safety data. We therefore paid particular attention to collect all treatment discontinuations, including their causes and to identify UTIs and genital mycosis. In instances where the reason for treatment withdrawal was not clearly stated, individual medical files were thoroughly reviewed to determine the underlying cause.

Our secondary objectives were to describe the evolution of blood pressure, proteinuria, and eGFR on treatment. Because the 2021 race-free CKD-Epidemiology Collaboration equation was found suboptimal for the care and follow-up of transplanted patients, we used the newly developed race-free kidney recipient–specific GFR equation.^17^^,^^18^ Nevertheless, we additionally estimated GFR using the race-free CKD-Epidemiology Collaboration equation, which is more largely utilized in current practice, in order to validate our primary results concerning graft function and the use of SGLT2i, as well as to facilitate comparison with prior studies.

### Statistical Analyses

Categorical variables were provided as absolute and relative frequencies. Depending on their distribution, continuous variables are described as the mean and SD or median and interquartile range. To compare categorical variables, we used a χ2 test or Fisher exact test where appropriate. To compare continuous variables, we used a Mann Whitney test or Kruskal Wallis test.

Cumulative incidence of treatment discontinuation was assessed by using a Kaplan Meyer model. Then, we included all factors possibly associated with treatment discontinuation into a time-dependent Cox regression model. All variables associated with treatment discontinuation (*P* < 0.05) were then included in the multivariate model. We additionally built cumulative incidence by eGFR and body mass index (BMI) and compared them using Gray’s test.

Next, we compared the evolution of eGFR, proteinuria, and blood pressure in patients with available data at initiation of SGLT2i and month-3, and initiation and month-6, using the Wilcoxon signed-rank test. A subgroup analysis was performed in patients with systolic and diastolic blood pressure available to determine SGLT2i’s effect in normotensive and hypertensive patients. The change in proteinuria levels after initiation was specifically conducted in proteinuric patients (proteinuria > 500 mg/d or urine protein-to-creatinine ratio > 500 mg/g), as well as in diabetic and nondiabetic KTRs. All tests were 2-sided, and *P* < 0.05 was considered significant. All statistical tests were performed with R Studio software (R Studio, version 1.2.1335).

## Results

### Baseline Characteristics

We included all consecutive 347 KTRs who started SGLT2i. Initiation occurred mostly (87%) after the first posttransplantation year. Baseline characteristics are shown in Table 1. Dapagliflozin was used in 335 patients (96.6%), whereas empagliflozin was given in others. At initiation of SGLT2i, eGFR ranged between 30 and 60 ml/min per 1.73 m^2^ in 75.8% of patients and <30 ml/min per 1.73 m^2^ in 8.4% of patients. More than two-thirds of the KTRs received ACE inhibitors (*n* = 132, 38.0%) or ARBs (*n* = 126, 36.3%), whereas 13 patients received a combination (3.7%). The most frequent immunosuppressive regimen consisted of a combination of calcineurin inhibitor and mycophenolate mofetil, with about half maintained on long-term corticotherapy.

**Table 1:** Baseline characteristics

Characteristics	Great Astre Cohort, (*N* = 347)
Clinical data at kidney transplantation	
First kidney transplant (%)	334 (96.3)
Preemptive kidney transplant (%)	40 (11.5)
Deceased donor (%)	295 (85.0)
Multiorgan transplantation (%)	5 (1.4)^a^
Induction with rATG (%)	109 (31.4)
Induction with basiliximab (%)	194 (55.9)
Coronary disease (%)	52 (15.0)
Heart failure (%)	23 (6.6)
Stroke (%)	15 (4.3)
Peripheral artery disease (%)	29 (8.4)
Hypertension (%)	291 (83.9)
Arrhythmia (%)	265 (76.4)
Clinical and biological data at initiation of SGLT2i	
Age (yrs)	62.6 [52.1–69.5]
Male (%)	265 (76.4)
Time after transplantation (yrs)	6.7 [2.8–13.5]
Diabetes mellitus (%)	226 (65.1%)
Weight (kg)	82.0 [72.0–91.4]^b^
BMI (kg/m^2^)	27.8 [24.5–32.0]^b^
eGFR (ml/min per 1.73 m^2^)	44.8 [37.1–54.5]
Serum creatinine (μmol/l)	142 [113–173]
Proteinuria (mg/d)^c^	530 [220–1546]^b^
Systolic blood pressure (mm Hg)	143 [132–155]^b^
Diastolic blood pressure (mm Hg)	81 [73–89]^b^
Immunosuppressive regimen at initiation of SGLT2i	
Calcineurin inhibitor (%)	261 (75.2)
Mycophenolate mofetil or sodium (%)	316 (91.1)
Steroids (%)	185 (53.3)
mTor inhibitors (%)	38 (11.0)
Belatacept (%)	14 (4.0)
Antihypertensive treatment at initiation of SGLT2i	
ACE inhibitors or ARBs (%)	245 (70.6)
Diuretic (%)	140 (40.4)
Calcium channel blocker (%)	202 (58.2)
Beta-blockers (%)	223 (64.3)
Alpha-blockers (%)	59 (17.0)
Centrally acting antihypertensive drugs (%)	33 (9.5)
Hypolipemiant treatment at initiation of SGLT2i	
Statin (%)	224 (64.6)
Ezetimibe (%)	48 (13.8)

ACE, angiotensin-converting enzyme; ARBs, angiotensin-receptor blockers; BMI, body mass index; eGFR, estimated glomerular filtration rate; rATG, rabbit antithymocyte globulin; SGLT2i, sodium-glucose cotransporter-2 inhibitor.

a *n* = 2 heart and kidney, *n* = 2 pancreas and kidney, *n* = 1 liver and kidney.

b Missing data (20 for weight, 29 for BMI, 74 for proteinuria, 38 for blood pressure).

c When data in mg/d was missing, it was substituted by urine protein-to-creatinine ratio in mg/g.

Most of the differences in the baseline characteristics between diabetic and nondiabetic patients were observed at the initiation of SGLT2i (Supplementary Table S1). The diabetic patients exhibited a higher BMI, better renal function, and lower levels of proteinuria, which may reflect the fact that diabetes was the primary indication for treatment in some cases. Of the 226 diabetic KTRs, 51.3%, 46.0%, and 2.6% had posttransplant diabetes mellitus, pretransplant type 2 diabetes, and pretransplant type 1 diabetes, respectively. Data on hemoglobin A1C was available in 130 patients at initiation of SGLT2i. Median hemoglobin A1C at initiation of SGLT2i was 7.5% (6.7–8.3). A vast majority received at least 1 antidiabetic treatment at initiation of SGLT2i (96.5%): metformin (56.2%), insulin (37.2%), dipeptidyl peptidase 4 inhibitors (26.1%). and glucagon-like peptide 1 agonists (14.6%).

Importantly, we were not able to give the indication of SGLT2i, even though baseline characteristics indirectly provide information about them (diabetes mellitus, proteinuria, cardiac disease). Thus, it is interesting to note that patient profiles were fairly homogeneous across centers (Supplementary Table S2).

Median follow-up duration was 12.0 (0.1–44.8) months. During this period, 2 patients returned to dialysis because of progression of chronic graft dysfunction. No death was reported.

### Safety Data

Ninety patients (25.9%) experienced at least 1 adverse event (Table 2). Graft dysfunction was reported in 4.9 % of patients, leading to SGLT2i withdrawal. All graft losses occurred in patients who underwent a preexisting severe and rapidly progressive graft dysfunction. Nevertheless, we could not clearly distinguish between acute kidney disease (AKI) occurring after the introduction of SGLT2i and preexisting graft dysfunction that continued to evolve, possibly even more quickly.

**Table 2:** Adverse events

Adverse events	Great Astre cohort (*N* = 347)	Diabetic patients (*n* = 226)	Nondiabetic patients (*n* = 121)	Leading to SGLT2i withdrawal (*n* = 54)
Graft dysfunction, n (%)	17 (4.9)	10 (4.4)	7 (5.8)	17 (100.0)
Infectious adverse events				
Lower UTI, n (%)	16 (4.6)	13 (5.8)	3 (2.5)	3 (18.8)
Pyelonephritis, n (%)	7 (2.0)	5 (2.2)	2 (1.7)	3 (42.9)
Genital infection, n (%)	2 (0.6)	0 (0.0)	2 (1.7)	0 (0.0)
SARS-CoV-2 infection, n (%)	22 (6.3)	17 (7.5)	5 (4.1)	1 (4.6)
Other pulmonary infection, n (%)	6 (1.7)	6 (2.7)	0 (0.0)	1 (16.7)
Digestive infection, n (%)	6 (1.7)	4 (1.8)	2 (1.7)	1 (16.7)
CMV primary infection, n (%)	4 (1.1)	4 (1.8)	0 (0.0)	1 (25.0)
Other infection, n (%)	5 (1.4)	4 (1.8)	1 (0.8)	5 (100.0)
BKV viremia, n (%)	2 (0.6)	2 (0.9)	0 (0.0)	1 (50.0)
Shingles, n (%)	2 (0.6)	2 (0.9)	0 (0.0)	0 (0.0)
Digestive symptoms, n (%)	12 (3.5)	7 (3.1)	5 (4.1)	5 (41.7)
Other adverse events				
Dysuria, n (%)	1 (0.3)	0 (0.0)	1 (0.8)	1 (100.0)
Ketoacidosis, n (%)	1 (0.3)	1 (0.4)	0 (0.0)	1 (100.0)
Hypoglycemia, n (%)	1 (0.3)	1 (0.4)	0 (0.0)	0 (0.0)
Pancreatitis, n (%)	1 (0.3)	1 (0.4)	0 (0.0)	0 (0.0)
Stroke, n (%)	3 (0.9)	3 (1.3)	0 (0.0)	0 (0.0)
Myocardial infarction, n (%)	1 (0.3)	1 (0.4)	0 (0.0)	0 (0.0)
Veinous thrombosis, n (%)	1 (0.3)	1 (0.4)	0 (0.0)	0 (0.0)
Gout, n (%)	2 (0.6)	1 (0.4)	1 (0.8)	1 (50.0)
Amputation, n (%)	1 (0.3)	1 (0.4)	0 (0.0)	0 (0.0)
Hypotension, n (%)	2 (0.6)	2 (0.9)	0 (0.0)	1 (50.0)
Dizziness, n (%)	1 (0.3)	0 (0.0)	1 (0.8)	1 (100.0)
Oedema, n (%)	1 (0.3)	1 (0.4)	0 (0.0)	1 (100.0)
Hyperkaliemia, n (%)	1 (0.3)	1 (0.4)	0 (0.0)	0 (0.0)
Basocellular tumor, n (%)	3 (0.9)	3 (1.3)	0 (0.0)	0 (0.0)
Spinocellular tumor, n (%)	2 (0.6)	1 (0.4)	1 (0.8)	0 (0.0)

AKI, acute kidney injury; BKV, BK virus; CMV, cytomegalovirus; SGLT2i, sodium-glucose cotransporter-2 inhibitor; UTI, urinary tract infection.

The most frequent adverse event was UTI, recorded in 23 patients (6.6%). They were mostly lower UTI that hardly led to SGLT2i discontinuation. Seven patients (2% of total patients) reported pyelonephritis, whereas 3 of them had already presented at least 1 pyelonephritis after their transplantation. It was difficult to determine whether UTIs were related to treatment. However, we noticed that after SGLT2i discontinuation for UTI in 6 patients, 4 KTRs presented with other episodes. In other 17 patients who were continuing SGLT2i despite a first UTI episode, 4 experienced another symptomatic infection during the follow-up. Genital mycosis were rare (0.6 %) and were exclusively reported in diabetic patients. No Fournier’s gangrene was reported. The other intercurrent infections are reported in Table 2, which primarily consisted of respiratory and digestive infections. Notably, one-third of reported digestive symptoms were attributable to gastrointestinal infections, with diarrhea being the most common manifestation. Digestive infections were due to *Campylobacter*, norovirus, and *Clostridium difficile* in 3, 2, and 1 patient, respectively. Respiratory infections were mostly because of SARS-CoV-2.

Although SGLT2i are contraindicated in patients with type 1 diabetes in France, dapagliflozin was introduced in 6 KTRs with type 1 diabetes. It was also stopped for this reason in 3 of these patients in the absence of adverse event. The only case of ketoacidosis occurred in a patient with type 2 diabetes.

One patient underwent a limb amputation considered because of a preexisting infectious arthritis (SGLT2i was continued in this KTRs), and 3 patients experienced stroke. All strokes occurred in hypertensive and insulin-requiring patients with diabetes; none led to SGLT2i discontinuation.

### Treatment Discontinuation

**Figure 1:**
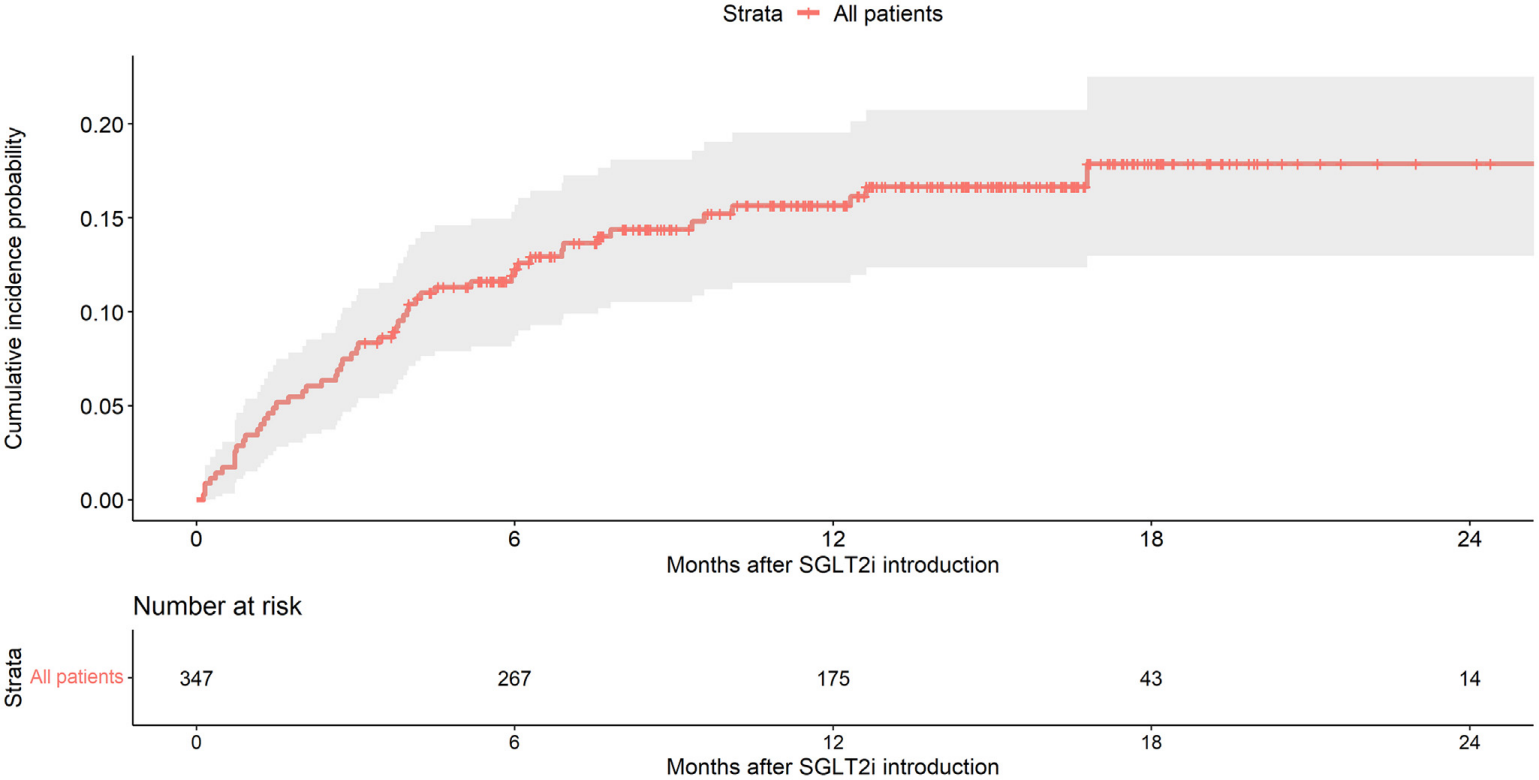
Cumulative incidence of SGLT2 inhibitor discontinuation in the whole cohort *(N* = 347). SGLT2i, sodium glucose cotransporter 2 inhibitor.

**Figure 2:**
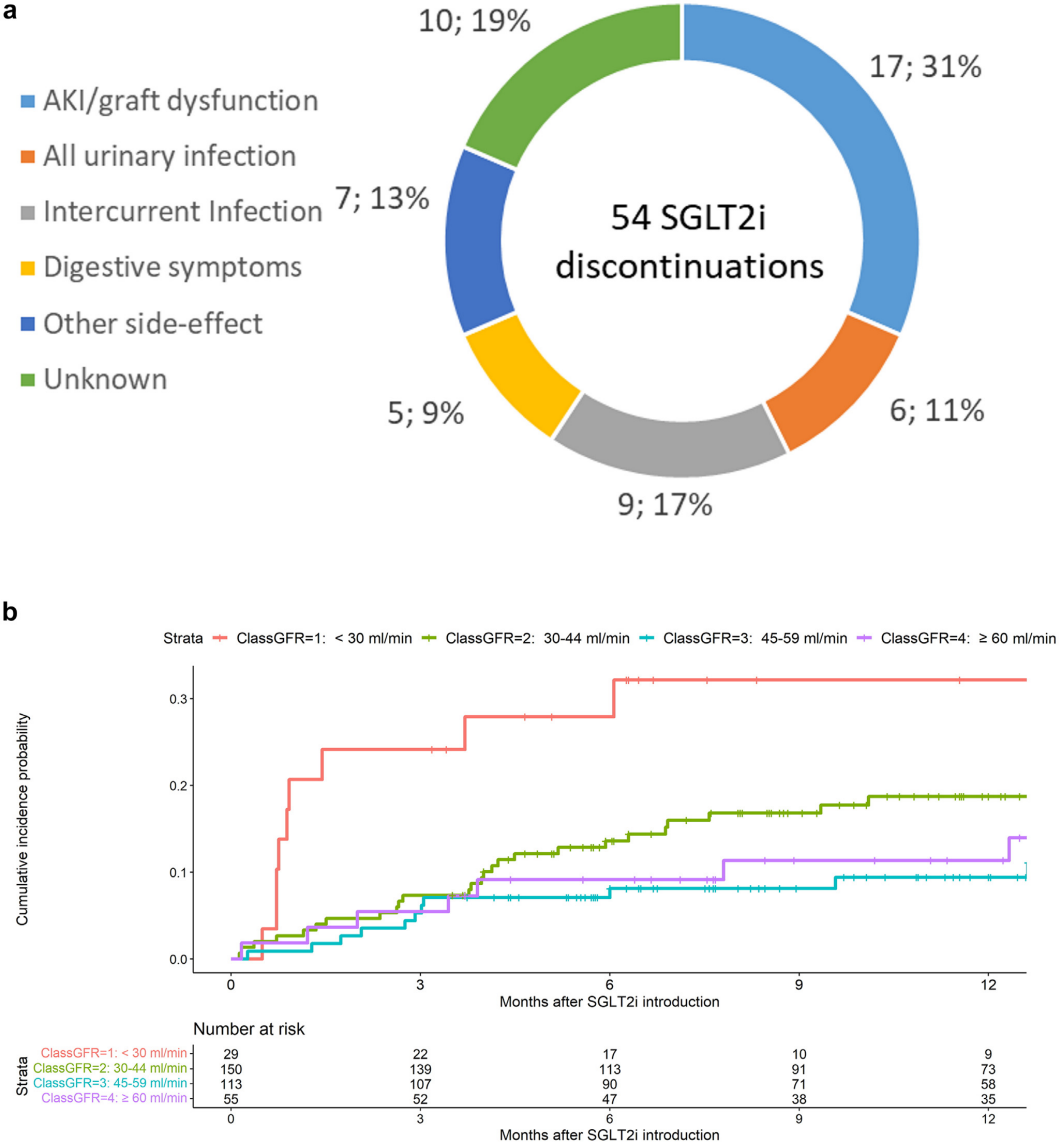
Causes of SGLT2 inhibitors discontinuation and incidence by graft function. (a) Pie chart of causes leading to SGLT2i discontinuation (*n* = 54). (b) Comparison of cumulative incidence of SGLT2i discontinuation by eGFR classes (< 30 ml/min per 1.73 m^2^, 30 to 44 ml/min per 1.73 m^2^, 45 to 59 ml/min per 1.73 m^2^, and > 60 ml/min per 1.73 m^2^). AKI, acute kidney injury; eGFR, estimated glomerular filtration rate; SGLT2i, sodium glucose cotransporter 2 inhibitor. eGFR is expressed in ml/min per 1.73 m^2^.

SGLT2i discontinuation was reported in 54 of the 347 patients over the follow-up period. Cumulative incidence of discontinuation was 7.8% (95% confidence interval: 5.4–11.2), 12.3% (95% confidence interval: 9.0–16.3] and 15.6% (95% confidence interval: 12.1–20.2) at 3, 6, and 12 months, respectively (Figure 1). The causes of discontinuation were led by graft dysfunction, reported in 4.9 % of the whole cohort, which translates to 32% of all causes of discontinuation (Figure 2a). Infectious causes of treatment discontinuation other than UTIs consisted of 1 norovirus infection, 1 SARS-CoV-2 infection, 1 primary cytomegalovirus infection, 1 septic shock, and 5 nonspecified infectious causes. In total, 17% of discontinuations were caused by intercurrent infections, which occurred significantly later than other causes (*P* = 0.008). We analyzed several parameters in a univariate model to determine if they were associated with SGLT2i withdrawal (Table 3). We found no association with the baseline comedication (ACE inhibitors or ARBs, diuretics, calcineurin inhibitors) or with the baseline comorbidities (diabetes mellitus or high blood pressure). Only initial eGFR and BMI were associated with SGLT2i discontinuation in the univariate analysis (*P* = 0.047 and *P* = 0.020, respectively). Hazard ratios were not modified after adjustment, even though only BMI remained significantly associated with SGLT2i discontinuation. Regarding BMI, we did not observe a gradual risk of SGLT2i discontinuation from KTRs with the highest BMI (≥ 35 kg/m^2^) to those with the lowest BMI (< 25 kg/m^2^) (Supplementary Figure S1).

**Table 3:** Parameters associated with SGLT2 inhibitor discontinuation

Parameters	Univariate analysis			Multivariate analysis		
	HR	95% CI	*P*-value	HR	95% CI	*P*-value
Time since Tx (per month)	1	0.999–1	0.777			
BMI (per kg/m^2^)	0.934	0.882–0.990	0.020	0.934	0.881–0.990	0.022
SBP (per mm Hg)	1.002	0.987–1.016	0.834			
DBP (per mm Hg)	0.998	0.974 –1.022	0.853			
Age (per yr)	1.023	0.999–1.047	0.064			
Sexe	1.066	0.619–1.835	0.626			
eGFR (per ml/min per 1.73 m^2^)	0.978	0.957–0.999	0.047	0.979	0.956–1.003	0.086
Diabetes mellitus	1.179	0.656–2.117	0.581			
CNI	0.826	0.455–1.498	0.529			
Tacrolimus	0.884	0.662–1.931	0.700			
Tacrolimus C0	1.167	0.680–1.079	0.188			
Ciclosporin	1.487	0.339–1.337	0.258			
Ciclosporin C0	1.008	0.968–1.017	0.516			
Diuretic	0.938	0.619–1.835	0.817			
Loop diuretic	1.300	0.317–5.334	0.716			

BMI, body mass index; CI, confidence interval; CNI, calcineurin inhibitor; C0, trough level; DBP, diastolic blood pressure; eGFR, estimated glomerular filtration rate; HR, hazard ratio; SBP, systolic blood pressure; Tx, transplantation.

To better investigate how initial eGFR predicted SGLT2i discontinuation, we categorized patients into 4 eGFR strata: < 30 ml/min per 1.73m^2^, 30 to 44.9 ml/min per 1.73 m^2^, 45 to 59.9 ml/min per 1.73 m^2^, and ≥ 60 ml/min per 1.73 m^2^. The incidence of SGLT2i discontinuation was different between groups (Figure 2b, *P* = 0.009). Thus, the incidence of SGLT2i discontinuation was higher in KTRs with eGFR < 45 ml/min per 1.73 m^2^ (*P* = 0.03) and even more in KTRs with eGFR < 30 ml/min per 1.73 m^2^ with about 30% of treatment withdrawal after 6 months (*P* = 0.003) (Supplementary Figure S2). We underscore that 40% of discontinuations were because of graft dysfunction in KTRs with eGFR < 45 ml/min per 1.73 m^2^, compared with 10.5% in the others. The race-free CKD-Epidemiology Collaboration formula was less effective in stratifying the risk of SGLT2i discontinuation according to graft function, despite the cumulative incidence curves exhibiting similar aspect (Supplementary Figure S3). Nonetheless, we identified a higher incidence of SGLT2i discontinuation in patients with an eGFR < 30 ml/min/1.73 m² was noticed, irrespective of the GFR estimation formula (Supplementary Figure S3).

### Evolution of Renal Function, Proteinuria, and Blood Pressure Over 6 Months

We reported on creatinine levels, eGFR, proteinuria, and blood pressure in KTRs who remained on SGLT2i in Table 4, as well as in patients with and without diabetes. We found a slight reduction in eGFR at 3 and 6 months (*P* < 0.0001), irrespective of diabetes status. It was similarly observed using the CKD-Epidemiology Collaboration eGFR (Supplementary Table S3). In contrast, the level of proteinuria appeared to distinguish patients with a further decline of graft function because eGFR remained steady after SGLT2i initiation in patients with a proteinuria < 500 mg/d (Supplementary Table S4).

**Table 4:** Evolution of renal function, proteinuria and blood pressure over the first 6 months

Whole cohort (*N* = 347)	*n*	At initiation (M0)	Month-3	*P*-value (M0–M3)	N	At initiation (M0)	Month-6	*P*-value (M0–M6)
eGFR (ml/min per 1.73 m^2^)	265	43.8 [IQR: 37.0–53.9]	43.1 [IQR: 34.1–53.1]	<0.0001	216	45.1 [IQR: 38.2–55.1]	43.5 [IQR: 35.9–53.6]	<0.0001
Serum creatinine (μmol/l)	265	146 [IQR: 116–174]	152 [IQR: 119–186]	<0.0001	216	140 [IQR: 112–172]	147 [IQR: 115–182]	<0.0001
Proteinuria (mg/d)^a^	168	748 [IQR: 312–1832]	637 [IQR: 276–1272]	0.0005	75	650 [IQR: 315–1448]	500 [IQR: 270–1094]	0.006
SBP (mm Hg)	223	143 [IQR: 132–155]	138 [IQR: 126–150]	<0.0001	181	143 [IQR: 132–155]	140 [IQR: 129–152]	0.022
DBP (mm Hg)	219	81 [IQR: 73–99]	78 [IQR: 69–85]	<0.0001	177	80 [IQR: 72–89]	78 [IQR: 70–84]	0.001
Diabetic patients (*n* = 226)								
eGFR (ml/min per 1.73 m^2^)	170	45.7 [IQR: 38.1–55.2]	44.7 [IQR: 34.6–54.0]	0.007	150	47.3 [IQR: 38.4–58.2]	46.4 [IQR: 38.0–55.5]	0.017
Serum creatinine (μmol/l)	170	141 [IQR: 112–169]	144 [IQR: 115–176]	0.004	150	137 [IQR: 108–167]	143 [IQR: 111–166]	0.020
Proteinuria (mg/d)^a^	101	530 [IQR: 230–1231]	426 [IQR: 230–1003]	0.004	49	530 [IQR: 290–1210]	430 [IQR: 290–860]	0.136
SBP (mm Hg)	143	143 [IQR: 132–155]	138 [IQR: 127–150]	0.007	123	142 [IQR: 131–155]	140 [IQR: 128–152]	0.201
DBP (mm Hg)	139	80 [IQR: 71–87]	75 [IQR: 69–83]	0.002	119	79 [IQR: 70–88]	78 [IQR: 70–84]	0.023
Nondiabetic patients (*n* = 121)								
eGFR (ml/min per 1.73 m^2^)	95	42.3 [IQR: 35.8–51.5]	40.6 [IQR: 33.4–49.2]	0.0004	66	42.9 [IQR: 35.9–51.8]	39.0 [IQR: 33.1–46.7]	<0.0001
Serum creatinine (μmol/l)	95	156 [IQR: 127–184]	162 [IQR: 132–205]	0.0005	66	156 [IQR: 122–179]	167 [IQR: 135–205]	<0.0001
Proteinuria (mg/d)^a^	67	1274 [IQR: 420–2487]	930 [IQR: 461–1784]	0.029	26	916 [IQR: 364–1865]	716 [IQR: 223–1333]	0.016
SBP (mm Hg)	80	145 [IQR: 134–158]	139 [IQR: 126–150]	0.0002	58	145 [IQR: 136–159]	142 [IQR: 131–151]	0.025
DBP (mm Hg)	80	84 [IQR: 78–91]	79 [IQR: 70–87]	0.0002	58	84 [IQR: 79–91]	79 [IQR: 70–87]	0.011

DBP, diastolic blood pressure; eGFR, estimated glomerular filtration rate; IQR, interquartile range; SBP, systolic blood pressure.

a Urinary protein-to-creatinine ratio expressed as mg/g was used in absence of 24-hour urine collection.

**Figure 3:**
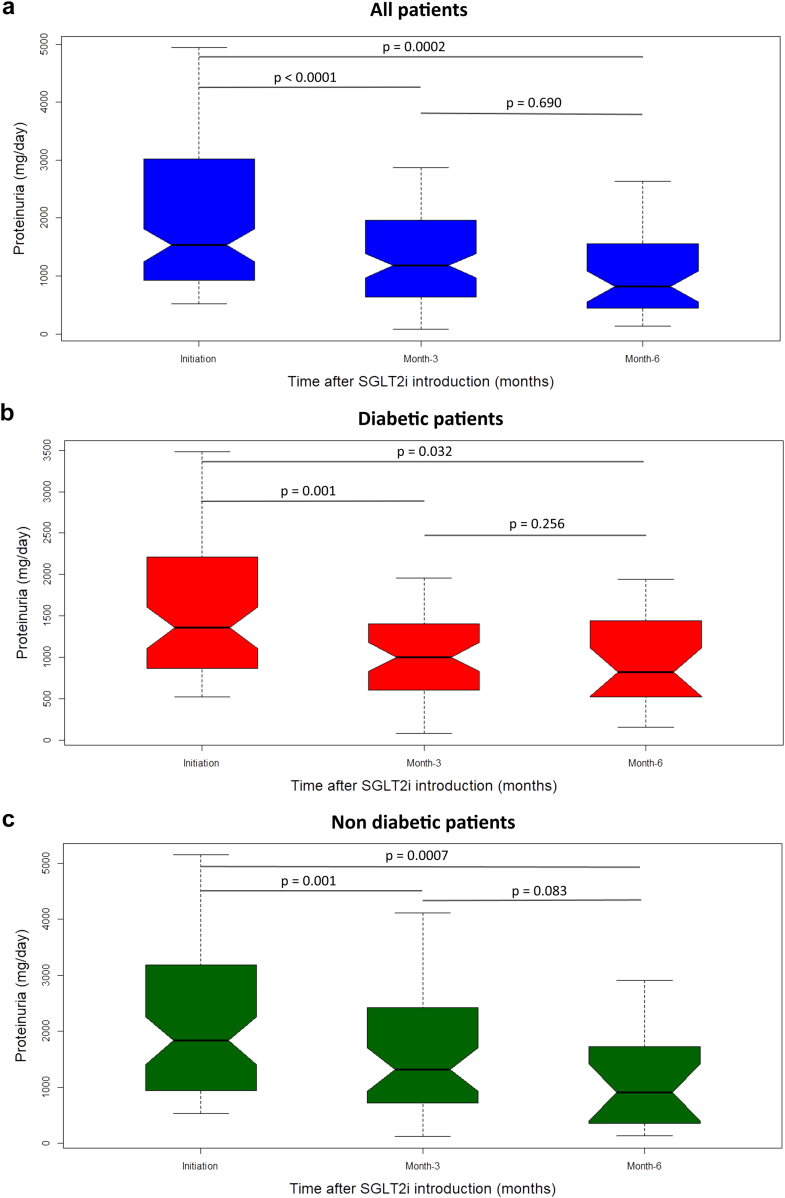
Evolution of proteinuria in proteinuric patients over 6 months follow-up. (a) In all proteinuric patients (at initiation: *n* = 99, at 3 months: *n* = 99, and at 6 months: *n* = 38); (b) in proteinuric diabetic patients (at initiation: *n* = 51, at 3 months: *n* = 51, and at 6 months: *n* = 22); (c) proteinuric nondiabetic patients (at initiation: *n* = 48, at 3 months: *n* = 48, and at 6 months: *n* = 16). SGLT2i, sodium glucose cotransporter 2 inhibitor.

**Figure 4:**
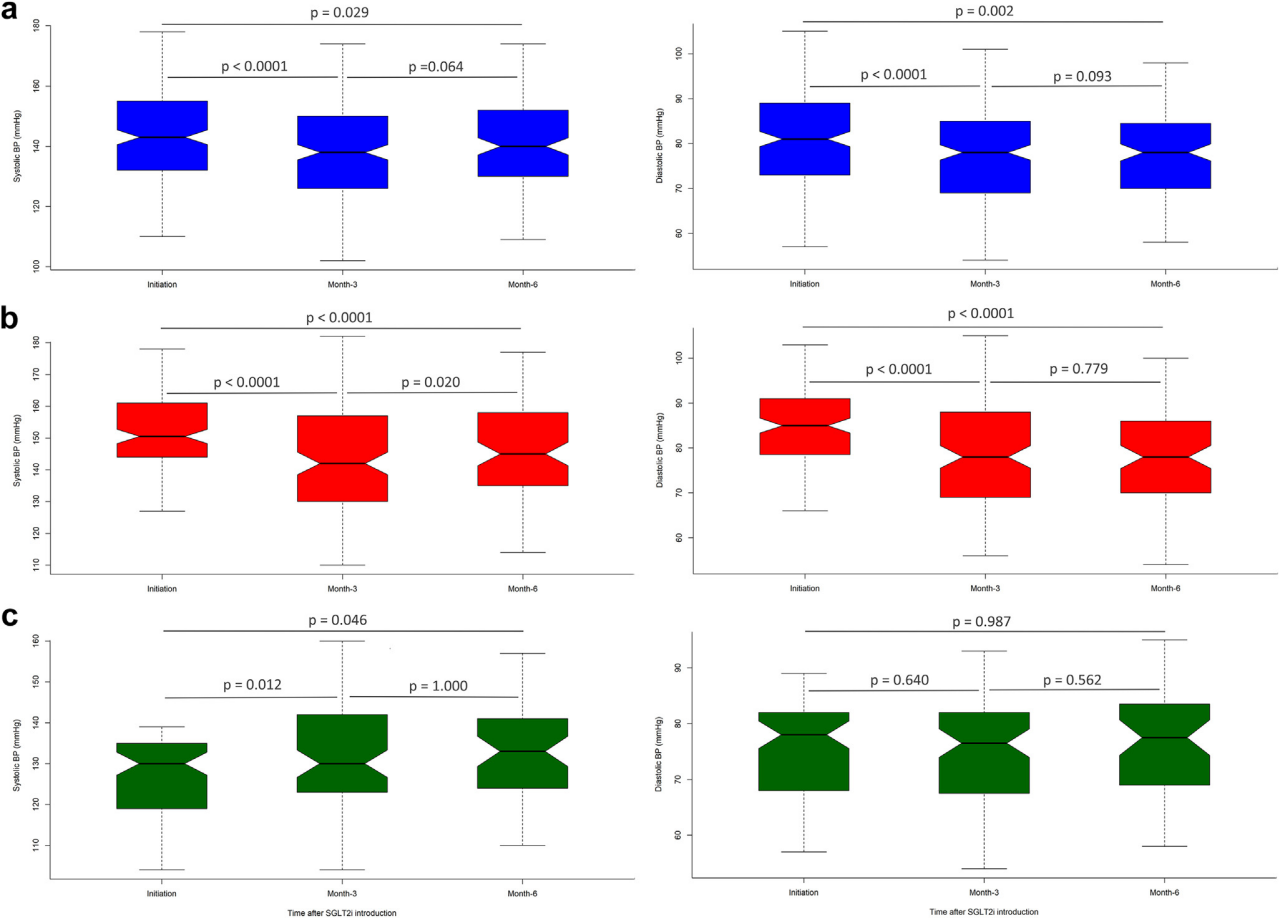
Evolution of systolic and diastolic blood pressure over 6 months follow-up. Systolic blood pressure (left panel) and diastolic blood pressure (right panel) are presented in (a) the whole cohort (at initiation: *n* = 223, at 3 months: *n* = 223, and at 6 months: *n* = 149), (b) in patients with baseline BP > 140/90 mm Hg (at initiation: *n* = 142, at 3 months: *n* = 142, and at 6 months: *n* = 96), and (c) in patients with baseline BP < 140/90 mm Hg (at initiation: *n* = 81, at 3 months: *n* = 81, and at 6 months: *n* = 53). BP, blood pressure; DBP, diastolic blood pressure; SBP, systolic blood pressure; SGLT2i, sodium-glucose cotransporter-2 inhibitor.

A 15% reduction in proteinuria was observed 3 months after SGLT2i initiation and remained significant over the first 6 months. In 138 patients with proteinuria > 500 mg/d, the reduction in proteinuria was observed in the first 3 months and reached 36% at 6 months (*P* < 0.0001) (Figure 3a). The antiproteinuric effect was similarly noticed in both proteinuric patients with diabetes (*n* = 70, *P* = 0.032) and nondiabetic patients (*n* = 68, *P* = 0.0007). (Figure 3b and c). We noted a reduction in systolic and diastolic blood pressure of about 5 and 3 mm Hg (*P* < 0.00001), respectively, after 3 months of SGLT2i use. The antihypertensive effect remained significant at 6 months, especially in nondiabetic patients (Table 4). We then stratified KTRs by initial blood pressure to investigate the reduction in blood pressure in hypertensive and normotensive KTRs. In patients with blood pressure ≥ 140/90 mm Hg, the systolic blood pressure decreased from 156 ± 16 mm Hg to 146 ± 168 mm Hg over the first 6 months, whereas SGLT2i had no antihypertensive effect in patients with blood pressure < 140/90 mm Hg. (Figure 4b and c).

## Discussion

Our main objective was to provide reliable safety data on the use of SGLT2i in KTRs in real-life conditions. Patients’ characteristics were similar to the DAPA-CKD population regarding proportion of nondiabetic patients and renal function. Notably, almost all patients received dapagliflozin, the first approved SGLT2i for patients with CKD in France. We therefore provide original data from a large cohort with distinct characteristics, differing from previous studies in KTRs that primarily enrolled diabetic recipients with good graft function and were mostly treated with empagliflozin.

First, we found that KTRs would be more prone to develop side effects leading to treatment discontinuation in comparison to nontransplanted patients. Indeed, 12.7% and 16.9% of CKD patients stopped dapagliflozin and empagliflozin over 24 months in the DAPA-CKD and EMPA-KIDNEY studies, respectively; whereas we reported 15.6% of discontinuation during a 1-year follow-up. This discontinuation rate was close to the 11.8%, 1-year withdrawal rate found in diabetic KTRs.^16^ In our study, treatment discontinuation occurred most often during the first 6 months following initiation. As expected, graft dysfunction and urinary infections represented 2 of the leading causes of SGLT2i discontinuation but occurred in only 23 patients (6.6 % of the whole cohort). Other causes were intercurrent infections that probably led to SGLT2i suspension by clinicians as commonly done with other medications that increase the risk of AKI in infectious settings (e.g., ACE inhibitors, ARBs, and diuretics), even though we were not able to provide reliable data about resumption of treatment. Digestive symptoms responsible for SGLT2i discontinuation were diverse, with diarrhea identified as the most common side effect. It is interesting to compare our results with the Spanish cohort that enrolled 339 diabetic patients, with a lower incidence of discontinuation because of AKI.^16^ This difference is probably related to the lower graft function of our cohort (44 vs. 58.4 ml/min per 1.73 m^2^). Indeed, they reported that AKI was more frequent in patients with eGFR < 40 ml/min per 1.73 m^2^, as we found that KTRs with eGFR < 45 ml/min per 1.73 m^2^ had an increased risk of SGLT2i discontinuation for graft dysfunction. Whether the higher proportion of patients treated by ACE inhibitors or ARBs in our cohort could additionally contribute to increased incidence of AKI may be debated, because it has been demonstrated that SGLT2i protect patients from AKI in several meta-analysis.^19^, ^20^, ^21^ Interestingly, in DAPA-CKD, including nontransplanted patients treated with ACE inhibitors or ARBs, discontinuation rates were higher in patients with CKD stage 4 versus CKD stage 2 or 3; however, the rates were similar in dapagliflozin versus placebo in both CKD 4 and CKD 2 or 3 group. Finally, we consider that early introduction of SGLT2i should be encouraged; that is, for eGFR > 45 ml/min per 1.73 m^2^. Finally, we observed that patients with the lowest BMI were more likely to discontinue treatment, which was not reported in previous studies. This finding may suggest greater fragility in these patients.

Second, we reported that UTI was the most frequent adverse event considered as related to SGLT2i. Incidence of UTI in diabetic KTRs treated by SGLT2i is known to be between 5.4% and 14%,^15^^,^^16^ whereas we identified that 7.9 % of our diabetic KTRs developed at least 1 UTI. Although we do not find a significantly lower incidence of UTI in nondiabetic KTRs (4.1%), the incidence of pyelonephritis (1.7%) was actually relatively close to incidence of severe UTI in nontransplanted patients that was between 1.5 and 1.7 %.^2^^,^^3^^,^^22^ We did not report any genital infection in nondiabetic KTRs, and <1% in diabetic KTRs, like in the Korean and Spanish studies.^15^^,^^16^ To interpret our findings accurately, it is important to acknowledge a potential selection bias. Few SGLT2i initiations took place in the early posttransplantation period, and investigators had collectively agreed to avoid prescribing SGLT2i to patients with urologic catheters.

Our study was not designed to evaluate the risk of graft failure or cardiovascular mortality, and we did not include a control group for comparison. Nevertheless, we report an improvement in blood pressure and proteinuria over the first 6 months of treatment, which was consistent across diabetic and nondiabetic patients. These results should be considered as a preliminary promising signal that SGLT2i could have a cardiovascular protective effect in KTRs regardless of their diabetic status, given the associations between proteinuria and uncontrolled hypertension with increased graft loss and worst survival outcomes.^9^^,^^10^^,^^23^ So far, 1 study showed an improved death-censored graft survival and patient survival in KTRs treated with SGLT2i, even though it was not confirmed after matching using a propensity score.^15^ A 5-year follow-up of our cohort will provide further insight into the efficacy of SGLT2i on cardiovascular and graft survival outcomes, as compared with matched patients in the ASTRE database. Although there is still no evidence about ACE inhibitors’ efficacy in graft and patient outcome in a large clinical trial of KTRs, our study highlights the potential of SGLT2i-based strategies for improving cardiovascular outcome, especially in recipients with the highest cardiovascular risk.

The first limitation of this study is the short-term follow-up that prevents us from drawing conclusions on long-term safety. However, treatment discontinuation occurred mainly in the first 6 months following initiation and was afterward mostly because of intercurrent infections unrelated to SGLT2i. The second limitation is the absence of independent committee adjudication. We therefore retrospectively interrogated investigators regarding all causes of discontinuations and infections. The extended follow-up of our ASTRE database with yearly visits will provide additional information regarding the resumption of SGLT2i and possibly recurrence of infections. The third limitation is the number of missing data, especially regarding proteinuria and blood pressure. Regarding blood pressure, an analysis of antihypertensive comedications, including possible dosage modifications during follow-up and home blood pressure monitoring should be more relevant.

In conclusion, this work supports the safety of prescribing SGLT2i to selected KTRs beyond the first transplantation year with characteristics similar to DAPA-CKD study, thus providing encouraging preliminary efficacy results on proteinuria and blood pressure.

## Disclosure

All the authors declared no competing interests.

## Supplementary Material

Supplementary File (PDF) **Figure S1.** Incidence of SGLT2 inhibitor discontinuation by initial body mass index. **Figure S2.** SGLT2 inhibitor discontinuation according to initial estimated glomerular filtration rate. **Figure S3.** Incidence of SGLT2 inhibitors discontinuation according to GFR estimated using race-free CKD-Epidemiology Collaboration formula. **Table S1.** Comparison of baseline characteristics between diabetic and nondiabetic patients. **Table S2.** Baseline characteristics of patients by centers. **Table S3.** Evolution of GFR (ml/min/1.73 m^2^) estimated by 2 race-free equations over the first 6 months. **Table S4.** Evolution of GFR (ml/min/1.73 m^2^) in proteinuric and nonproteinuric patients.

## Footnotes

Supplementary File (PDF)
**Figure S1.** Incidence of SGLT2 inhibitor discontinuation by initial body mass index. **Figure S2.** SGLT2 inhibitor discontinuation according to initial estimated glomerular filtration rate. **Figure S3.** Incidence of SGLT2 inhibitors discontinuation according to GFR estimated using race-free CKD-Epidemiology Collaboration formula. **Table S1.** Comparison of baseline characteristics between diabetic and nondiabetic patients. **Table S2.** Baseline characteristics of patients by centers. **Table S3.** Evolution of GFR (ml/min/m2) estimated by 2 race-free equations over the first 6 months. **Table S4.** Evolution of eGFR (ml/min/m2) in proteinuric and nonproteinuric patients.
