# Supplementary material for: Sodium-Glucose Cotransporter-2 Inhibitor in Diabetic and Nondiabetic Renal Transplant Recipients
Source: Kidney Int Rep. 2024 Nov 28;10(3):816–27. doi: 10.1016/j.ekir.2024.11.033 (PMC11993682; doi:10.1016/j.ekir.2024.11.033)

Table S1: Comparison of baseline characteristics between diabetic and non-diabetic patients

	**Diabetic patients (n=226)**	**Non diabetic patients (n=121)**	**p-value**
**Clinical data at kidney transplantation**			
Preemptive kidney transplant (%)	25 (11.4)	15 (13.2)	0.711
Deceased donor (%)	193 (85.4)	102 (84.3)	0.784
Multi-organ transplantation (%)	5 (2.3)	0 (0.0)	0.167
Induction with rATG Basiliximab (%)	67 (29.6)	42 (34.7)	0.333
Coronary disease (%)	37 (16.8)	15 (13.4)	0.323
Heart failure (%)	18 (8.2)	5 (4.5)	0.171
Stroke (%)	11 (5.0)	4 (3.6)	0.589
Peripheral artery disease (%)	22 (10.8)	7 (6.5)	0.205
Hypertension (%)	204 (92.3)	87 (77.0)	**<0.0001**
**Clinical and biological data at initiation of SGLT2i**			
Age (years)	64.0 [55.3 - 69.7]	57.9 [48.5-68.3]	**0.003**
Male (%)	177 (78.3)	88 (72.7)	0.243
Time after transplantation (years)	9.2[4.0 - 17.4]	5.7 [2.2-12.4]	**0.0004**
Weight (kg)	75.0 [63.0 - 84.0]	85 [75 - 97]	**<0.0001**
BMI (kg/m²)	29.4 [25.7 - 33.6]	25.6 [23.0 - 29.0]	**<0.0001**
eGFR (ml/min/1.73m²)	46.9 [38.6 - 56.4]	42.1 [35.4 - 50.7]	**0.002**
Serum creatinin (µmol/L)	138 [109 - 168]	157 [124 - 187]	**0.001**
Proteinuria (mg/day)*	421 [211 - 1404]	716 [320 - 1850]	**<0.0001**
Systolic blood pressure (mmHg)	142 [131 - 155]	145 [135 - 158]	0.300
Diastolic blood pressure (mmHg)	80 [70 - 88]	84 [78 - 90]	**0.002**
**Immunosuppressive regimen at initiation of SGLT2i**			
Calcineurin inhibitor (%)	174 (77.0)	87 (71.9)	0.360
Mycophenolate mofetil or sodium (%)	119 (52.7)	57 (47.1)	0.288
Steroids (%)	115 (50.9)	70 (57.9)	0.215
mTor inhibitors (%)	23 (10.2)	15 (12.4)	0.528
Belatacept (%)	6 (2.7)	8 (6.6)	0.074
**Anti-hypertensive treatment at initiation of SGLT2i**			
ACE inhibitors or ARBs (%)	79 (35.0)	47 (38.8)	0.473
Diuretic (%)	93 (41.2)	47 (38.8)	0.676
Calcium chanel blocker (%)	144 (63.7)	58 (47.9)	**0.005**
Beta-blockers (%)	150 (66.4)	73 (60.3)	0.263
Alpha-blockers (%)	39 (17.3)	20 (16.5)	0.863
Centrally acting antihypertensive drugs (%)	18 (8.0)	15 (12.4)	0.180
**Hypolipemiant treatment at initiation of SGLT2i**			
Statin (%)	160 (70.8)	64 (52.9)	**0.001**
Ezetimibe (%)	36 (15.9)	12 (9.9)	0.122

ACE: angiotensin converting enzyme, ARBs: angiotensin receptor blockers, BMI: body mass index, eGFR: estimated glomerular filtration rate, rATG: rabbit anti-thymocyte globulin, SGLT2i: sodium glucose cotransporter 2 inhibitor

** when data in mg/day was missing, it was substituted by proteinuria/creatininuria ratio in mg/g*

Table S2: Baseline characteristics of patients in all centres

Centre	Patients n (%)	Delay Tx-iSGLT2 (years)	BMI (kg.m²)	eGFR (ml/min/1.73m²)	Proteinuria (mg/day)	Diabetes mellitus n (%)
1	13 (3.7)	4.4 [2.8-7.2]	27.5 [25.4-33.2]	52.2 [44.3-56.8]	667 [387-1415]	10 (76.9)
2	8 (2.3)	12.9 [9.1-14.7]	26.2 [24.1-28.5]	48.7 [43.0-70.4]	716 [514-918]	6 (75.0)
3	2 (0.6)	12.6 [11.3-13.8]	27.1 [23.4-30.9]	47.0 [44.3-49.7]	811 [1125-249[illegible]	1 (50.0)
4	12 (3.5)	5.6 [1.4-12.3]	27.9 [27.1-33.6]	45.2 [41.6-57.8]	135 [115-379]	8 (66.7)
5	58 (16.7)	4.0 [0.7-10.6]	27.8 [23.9-32.5]	44.2 [38.5-53.4]	310 [190-540]	36 (62.1)
6	11 (3.2)	4.4 [2.8-5.2]	26.2 [24.6-29.4]	52.9 [38.4-57.7]	[illegible]271 [904-1334	4 (36.4)
7	19 (5.5)	11.2 [3.4-15.8]	27.3 [24.8-29.9]	48.7 [42.4-62.7]	200 [128-507]	19 (100.0)
8	36 (10.4)	9.3 [3.8-17.0]	27.4 [23.4-31.1]	39.4 [32.4-47.2]	[illegible]423 [201-2580	14 (38.8)
9	46 (13.3)	9.2 [4.6-17.6]	26.8 [24.1-29.7]	43.0 [33.8-53.9]	860 [370-1600]	19 (41.3)
10	26 (7.5)	8.3 [4.4-16.2]	28.6 [26.1-33.4]	45.6 [38.8-54.9]	521 [392-1255]	19 (73.1)
11	22 (6.3)	5.3 [4.0-11.6]	31.9 [27.1-34.8]	50.3 [37.3-59.7]	419 [129-1064]	15 (68.2)
12	29 (8.4)	5.2 [2.5-7.3]	29.6 [26.3-34.5]	49.6 [42.4-55.6]	314 [191-586]	28 (96.6)
13	65 (18.7)	6.9 [2.5-16.0]	27.0 [24.2-30.8]	42.3 [38.0-48.1]	884 [331-2073]	39 (60.0)

Delay Tx-iSGLT2: delay between transplantation and initiation of treatment

BMI: body mass index

eGFR: glomerular filtration rate estimated by race-free transplant specific formula

Table S3: Evolution of GFR (ml/min/m²) estimated by two race-free equations over the first 6 months.

Table S2: Evolution of GFR (ml/min/m²) estimated by two race-free equations over the first 6 months.

	N	At initiation (M0)	Month-3	P-value (M0-M3)	N	At initiation (M0)	Month-6	P-value (M0-M6)
**Whole cohort (n=347)**								
Transplant specific formula	265	43.8 [37.0-53.9]	43.1 [34.1-53.1]	**<0.0001**	216	45.1 [38.2-55.1]	43.5 [35.9-53.6]	**<0.0001**
CKD-EPI formula	265	43.9 [31.0-56.2]	43.2 [31.0-56.2]	**<0.0001**	216	45.9 [36.4-59.8]	43.5 [33.5-56.5]	**<0.0001**
**Diabetic patients (n=226)**								
Transplant specific formula	170	45.7 [38.1-55.2]	44.7 [34.6-54.0]	**0.007**	150	47.3 [38.4-58.2]	46.4 [38.0-55.5]	**0.017**
CKD-EPI formula	170	46.3 [36.6-59.5]	45.1 [32.3-58.5]	**0.0006**	150	48.5 [37.1-63.3]	47.1 [36.4-60.9]	**0.010**
**Non-diabetic patients (n=121)**								
Transplant specific formula	95	42.3 [35.8-51.5]	40.6 [33.4-49.2]	**0.0004**	66	42.9 [35.9-51.8]	39.0 [33.1-46.7]	**<0.0001**
CKD-EPI formula	95	40.7 [33.5-54.3]	38.8 [30.3-51.0]	**0.0003**	66	42.0 .(33.5-54.5]	36.7 [29.5-46.7]	**<0.0001**

Table S4: Evolution of GFR (ml/min/m²) in proteinuric and non-proteinuric patients

	N	At initiation (M0)	Month-3	P-value (M0-M3)	N	At initiation (M0)	Month-6	P-value (M0-M6)
**Protéinuric patients (n=138)**								
eGFR (ml/min/1.73m²)	113	40.6 [35.1-50.8]	39.7 [31.4-48.5]	**< 0.0001**	79	40.1 [34.8-50.2]	39.0 [31.7-47.4]	**< 0.0001**
Serum creatinine (µmol/L)	113	158 [123-186]	160 [129-209]	**< 0.0001**	79	157 [125-188]	164 [135-210]	**< 0.0001**
Protéinuria (mg/day or mg/g)	99	1540 [947-2998]	1180 [640-1957]	**< 0.0001**	43	1281 [905-1975]	820 [449-1554]	**< 0.0001**
SBP (mmHg)	99	146 [136-166]	142 [130-160]	**0.005**	68	146 [136-166]	141 [132-153]	**0.021**
DBP (mmHg)	97	84 [76-90]	78 [68-85]	0.064	66	82 [75-90]	78 [69-85]	0.064
**Patients without proteinuria (n=133)**								
eGFR (ml/min/1.73m²)	95	43.9 [38.0-53.8]	44.2 [35.9-51.9]	0.147	85	47.8 [39.0-58.4]	46.5 [37.7-54.3]	0.279
Serum creatinine (µmol/L)	95	145 [120-171]	148 [123-180]	0.218	85	133 [109-167]	141 [112-166]	0.371
SBP (mmHg)	86	140 [129-149]	135 [124-145]	0.071	74	141 [130-150]	140 [128-151]	0.846
DBP (mmHg)	85	80 [72-85]	78 [69-84]	**0.004**	74	79 [70-84]	78 [78-83]	0.142

eGFR: estimated glomerular filtration rate (race-free transplantation specific formula), SBP: systolic blood pressure, DBP: diastolic blood pressure;

** when data in mg/day was missing, it was substituted by proteinuria/creatininuria ratio in mg/g*

Figure S1: Incidence of SGLT2i discontinuation by initial body mass index (BMI, kg/m²)

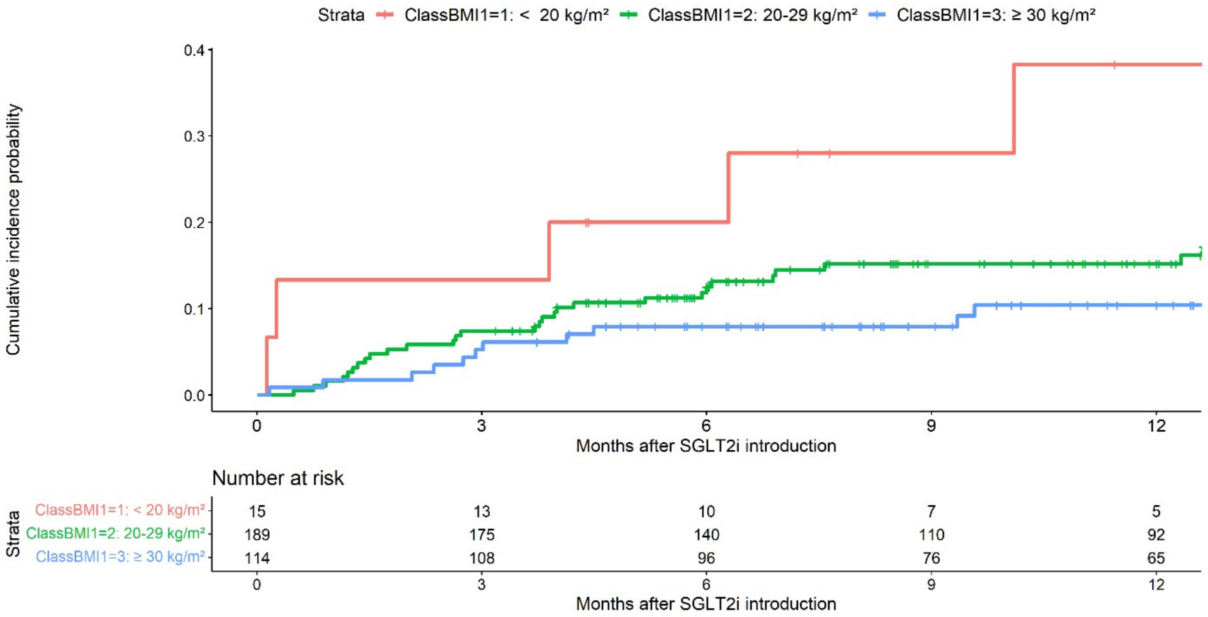

Figure S2: SGLT2 inhibitor discontinuation according to initial estimated glomerular filtration rate.

- Patients with eGFR < 45 ml/min/1.73m² vs. ≥ 45 ml/min ($p = 0.03$)

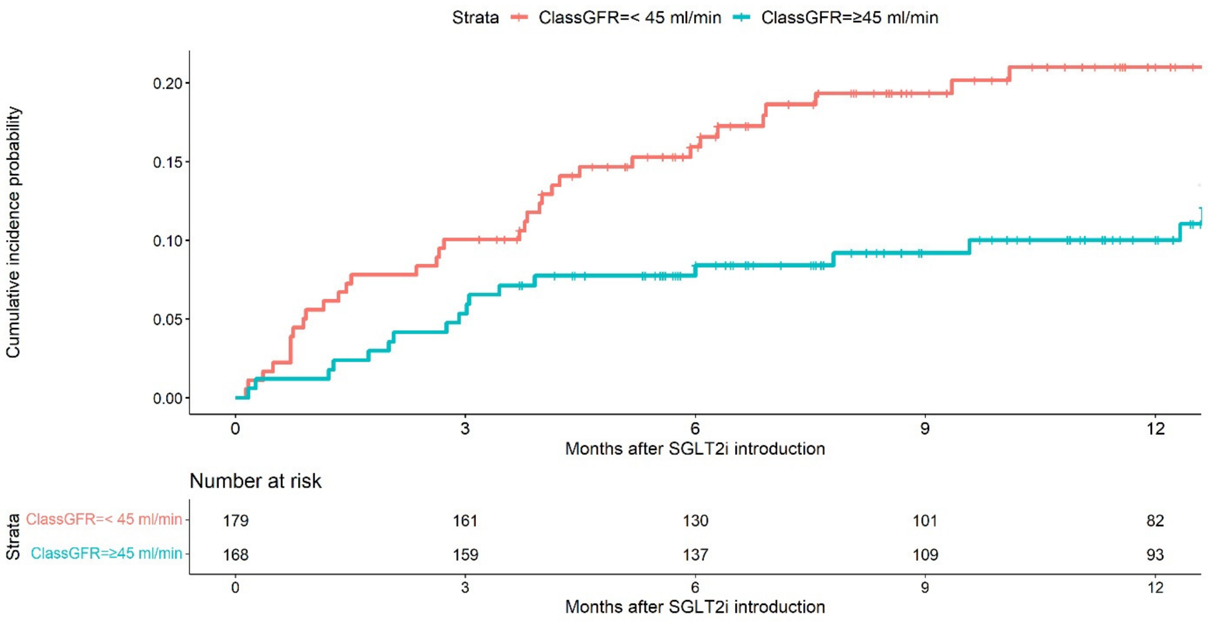

- Patients with eGFR < 30 ml/min/1.73m² vs. ≥ 30 ml/min ($p = 0.003$)

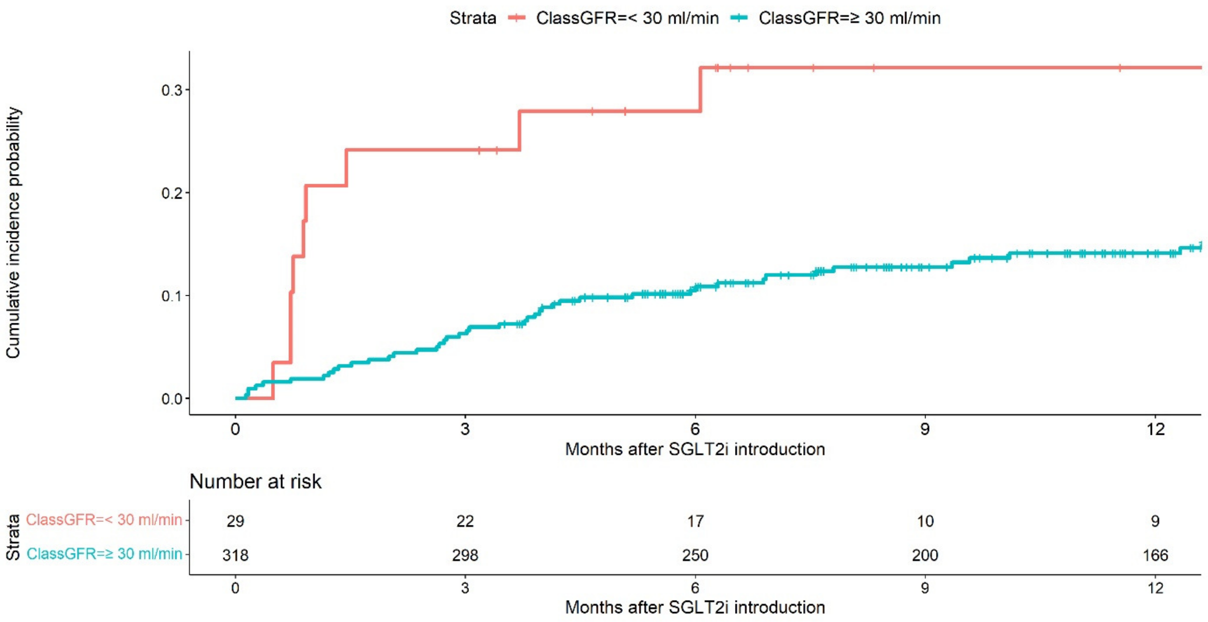

Figure S3: incidence of SGLT2 inhibitors discontinuation according to GFR estimated using race-free CKD-EPI formula .

A: Comparison of distribution of patients into 4 GFR classes estimated by race-free transplant specific or race-free CKD-EPI formula

		Race-free CKD-EPI 2021 equation			
		< 30 ml/min	30-44 ml/min	45-59 ml/min	≥ 60 ml/min
New race-free transplant specific equation	< 30 ml/min	29	23	0	0
	30-44 ml/min	0	122	3	0
	45-59 ml/min	0	5	86	0
	≥ 60 ml/min	0	0	24	55

B: Comparison of cumulative incidence of SGLT2i discontinuation by eGFR classes (< 30 ml/min/1.73m², 30 to 44 ml/min/1.73m², 45 to 59 ml/min/1.73m² and above 60 ml/min/1.73m²). No difference was observed between all 4 groups (p=0.2)

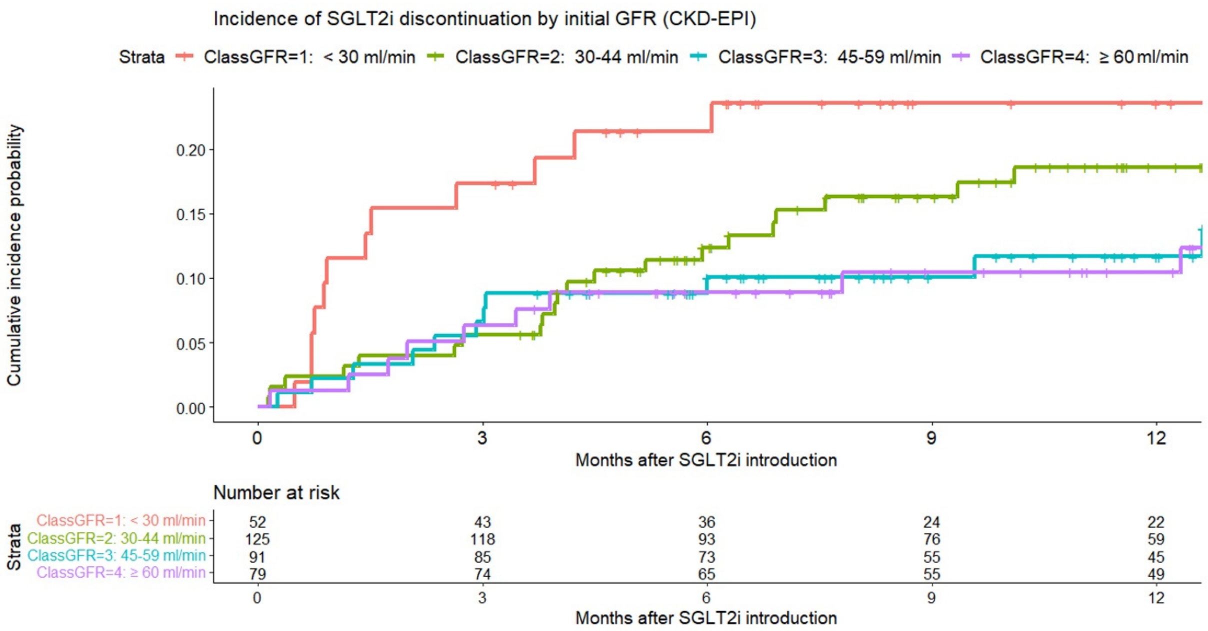

C: Comparison of cumulative incidence of SGLT2i discontinuation between patients with initial eGFR < 30 ml/min/1.73m² and ≥ 30 ml/min ($p = 0.05$)

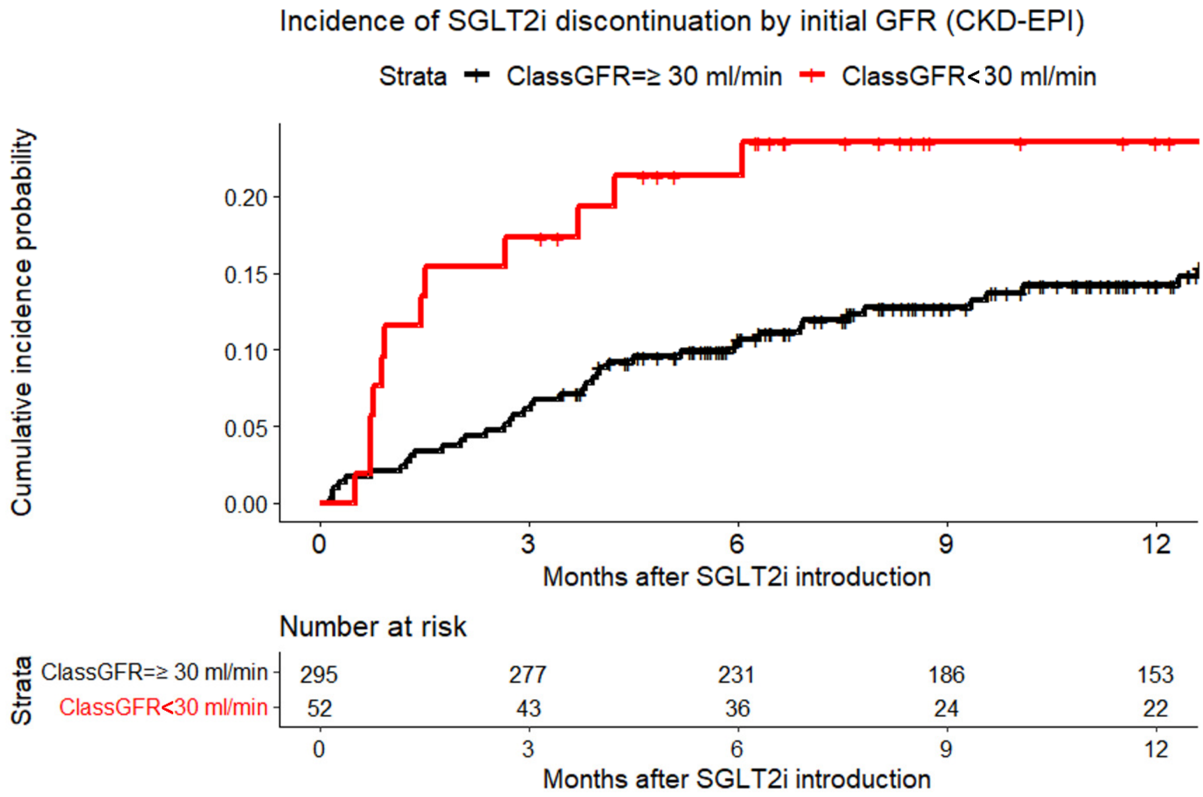

Supplement: Supplementary File (PDF) — Figure S1. Incidence of SGLT2 inhibitor discontinuation by initial body mass index. Figure S2. SGLT2 inhibitor discontinuation according to initial estimated glomerular filtration rate. Figure S3. Incidence of SGLT2 inhibitors discontinuation according to GFR estimated using race-free CKD-Epidemiology Collaboration formula. Table S1. Comparison of baseline characteristics between diabetic and nondiabetic patients. Table S2. Baseline characteristics of patients by centers. Table S3. Evolution of GFR (ml/min/1.73 m2) estimated by 2 race-free equations over the first 6 months. Table S4. Evolution of GFR (ml/min/1.73 m2) in proteinuric and nonproteinuric patients. [file mmc1.pdf]
